# Methylation status of DJ-1 in leukocyte DNA of Parkinson’s disease patients

**DOI:** 10.1186/s40035-016-0052-6

**Published:** 2016-03-31

**Authors:** Yuyan Tan, Li Wu, Dunhui Li, Xiaoli Liu, Jianqing Ding, Shengdi Chen

**Affiliations:** Department of Neurology, and Institute of Neurology, Ruijin Hospital Affiliated to Shanghai Jiao Tong University School of Medicine, Shanghai, 200025 China; Department of Neurology, Shanghai Ninth People’s Hospital Affiliated to Shanghai Jiao Tong University School of Medicine, Shanghai, 200011 China; Parkinson’s Disease Center, Beijing Institute for Brain Disorders, Beijing, 100069 China

**Keywords:** Parkinson’s disease, DJ-1, DNA methylation, Peripheral blood leukocytes

## Abstract

**Background:**

DJ-1 has been thought as a candidate biomarker for Parkinson’s disease (PD). It was found reduced in PD brains, CSF and saliva, although there were conflicting results. How DJ-1 expression may be regulated is not clear. Recently, blood-based DNA methylation represents a highly promising biomarker for PD by regulating the causative gene expression. Thus, in this study, we try to explore whether blood-based DNA methylation of DJ-1 could be used as a biomarker to differentiate PD patients from normal control (NC), and whether DNA methylation could regulate DJ-1 expression in a SH-SY5Y cell model.

**Methods:**

Forty PD patients and 40 NC were recruited in this study. DNA was extracted from peripheral blood leukocytes (PBLs). Methylation status of two CpG islands (CpG1 and CpG2) in promoter region of DJ-1 was explored by bisulfite specific PCR-based sequencing method. Methylation inhibitor 5-Aza-dC was used to treat SH-SY5Y cell line, DJ-1 level was detected in both mRNA and protein level.

**Results:**

CpG sites in these two CpG islands (CpG1 and CpG2) of DJ-1 were unmethylated in both PD and NC group. In SH-SY5Y cell model treated by methylation inhibitor, there was no significant change of DJ-1 expression in either mRNA level or protein level.

**Conclusions:**

Our results indicated that DNA methylation inhibitor didn’t alter DJ-1 gene expression in SH-SY5Y cell model, and DNA methylation of DJ-1 promoter region in PBLs level might not be an efficient biomarker for PD patients.

## Background

Parkinson’s disease (PD) is the second most common neurodegenerative disorders, pathologically characterized by a progressive degeneration of dopaminergic neurons and the presence of intracytoplasmic Lewy bodies (LBs). The mechanisms responsible for neuronal degeneration in PD are complex and remain to be fully elucidated.

Mutations of DJ-1 are linked to autosomal recessive early-onset PD [1]. Extensive studies showed that DJ-1 has neuroprotective functions in anti-oxidative stress, anti-inflammation, mitochondrial regulation [2]. Loss of DJ-1 function was shown to cause autosomal recessive PD [1]. In sporadic PD patients, the level of total DJ-1 protein was significantly reduced in the substantia nigra (SN) and CSF [3–5], although there were conflicting results [6]. These results suggested that lower DJ-1 level in PD patients may contribute to the pathogenesis of PD. However, the molecular mechanisms underlying the decreased DJ-1 level are not yet clear.

In recent years, epigenetic mechanisms such as DNA methylation, histone modification, chromatin remodeling and non-coding RNA regulation have been evidenced to play a role in regulating gene expression in neurodegenerative diseases [7–9]. DNA methylation at the 5-carbon position of cytosine residues located in dinucleotide CpG sites has the specific effect of reducing gene expression and unmethylated CpG sites are mostly linked to gene activation [10]. Reduced CpG island methylation in intron 1 of SNCA has been evidenced in PD brain tissue [11, 12], and hypomethylation of CpG was associated with increased a-synuclein expression [12]. Our previous results showed that the hypomethylation of SNCA in PD can also be detected in the blood-based DNA methylation [13]. Eliezer Masliah et al. identified concordant methylation alterations in brain and blood by investigating genome-wide DNA methylation in brain and blood samples from PD patients and NC [14]. These reports from both of Eliezer Masliah et al. and us indicated that DNA extracted from leukocytes in peripheral blood might be a potential effective noninvasive source for screening epigenetic biomarker for PD diagnosis. Until now, no data on the methylation status of DJ-1 has been reported. In our study, we aimed to detect whether there was differential DNA methylation of DJ-1 in the peripheral blood between PD and NC groups and determine whether DNA methylation of DJ-1 in peripheral blood could represent the reduced expression of DJ-1 in PD patients. In SH-SY5Y cell model we tested whether DNA methylation can regulate DJ-1 expression by using methylation inhibitor.

## Methods

### Subjects

Forty PD patients, diagnosed by the UK PD brain bank criteria, were enrolled from the Department of Neurology, Ruijin Hospital affiliated to Shanghai Jiao Tong University School of Medicine, Shanghai, China; 40 NC were recruited from our previous epidemiological studies. For each subject, 5 ml of blood samples and clinical data, such as name, gender, age, age of onset, duration, disease stage (H-Y stage) were collected. The PD and NC groups were well matched for age (PD: 63.7 ± 6.16 years; NC: 61.28 ± 9.21 years, *p* = 0.228) and gender (PD: 24/16; NC: 24/16). All the subjects have signed the informed consent form, and the study was approved by the Ruijin Hospital Ethics Committee, Shanghai Jiao Tong University School of Medicine.

### DNA extraction and bisulfite specific PCR-based sequencing method

Genomic DNA was extracted from peripheral blood according to the standard procedures. CPGPLOT (http://emboss.bioinformatics.nl/cgi-bin/emboss/cpgplot) was used to identify and plot CpG islands in DJ-1 promoter region. Primer3 (http://frodo.wi.mit.edu/) was used to design PCR bisulfite conversion-specific primers (Table 1).

**Table 1:** Primers for PCR assays

Primer	Sequence (5’ > 3’)	Tm (°C)	Amplicon (bp)
DJ-1- CpG1-methylation-PCR-F	GTTYGGGAGGTTTGGATTAGAGTT	56	170
DJ-1- CpG1-methylation-PCR-R	ACRACTCRATCCCACATAATACCC		
DJ-1- CpG2-methylation-PCR-F	TTGYGTAGTGTGGGGTTGAGG	58	141
DJ-1- CpG2-methylation-PCR-R	ACCRTCCAACACAAAAACACC		
DJ-1- CpG1-methylation-Seq-R	ACRACTCRATCCCACATAATACCC		
DJ-1- CpG2-methylation-Seq-R	ACCRTCCAACACAAAAACACC		
DJ-1- Realtime-F	CGGGGTGCAGGCTTGTAAA	58	150
DJ-1- Realtime-R	TCCGGTTTTCCTGCTCCTTC		

Bisulfite Specific PCR-based sequencing method was described in our previous report [13]. 1 μg DNA was treated with EZ DNA Methylation-GoldKit (ZYMO RESEARCH) following the manufacture’s protocol. After bisulfite conversion, unmethylated cytosines were converted to uracils, the converted product was purified followed by PCR amplification and sequencing. A 20 μl mixture was prepared for each reaction and included 1× HotStarTaq buffer, 2.0 mM Mg2+, 0.2 mM dNTP, 0.2 μM of each primer, 1 U HotStarTaq polymerase (Qiagen Inc.) and 1 μl template DNA. The cycling program was 95 °C for 15 mins; 11 cycles of 94 °C for 20s, 60–0.5 °C per cycle for 40 s, 72 °C for 1 min; 24 cycles of 94 °C for 20 s, 54 °C for 30 s, 72 °C for 1 min; 72 °C for 2 mins. 1U SAP and 6U Exo I were added into 8 μl PCR products for PCR purification. The mixtures were incubated at 37 °C for 60mins, followed by incubation at 70 °C for 10mins. In PCR amplification, the uracils were amplified as thymines, whereas 5-MeC residues were amplified as cytosines. The PCR products were purified to remove any primer dimers and sequenced on an ABI 3730 XL analyzer (Applied Biosystems and Life Technologies, USA). DNA methylation in the PCR target region was then read by scoring the remaining cytosine residues in the sequence. The degree of methylation at each CpG site from the direct sequencing profile was estimated by measuring the relative peak height of the cytosine (C) versus cytosine plus thymine (T) profile (C/(C + T)%). As this can only be regarded as semi-quantitative, the degree of methylation was expressed as 0, 25, 50, 75 or 100 %.

### Cell culture and treatment with 5-Aza-dC

SH-SY5Y cells (ATCC) were cultured in DMEM with 10 % FBS, 100 U/ml penicillin/streptomycin (Invitrogen) and maintained at 37 °C with 5 % CO2. SH-SY5Y cells were chemically treated with 5-Aza-dC (2.5 or 5 mM; Sigma). Real time PCR and Western blot were used to detect the mRNA and protein level of DJ-1. Total RNA was extracted from SH-SY5Y cells after 24 h 5-Aza-dC treatment by a standard method with TRIZOL Reagent (Invitrogen), and reverse-transcribed by using the PrimeScript® RT reagent Kit (Takara) according to the manufacture’s instruction. The mRNA levels were detected through real-time PCR by a standard method with Realtime PCR Master Mix (SYBR Green) kit (Takara). The primers for real-time PCR have been summarized in Table 1. We extracted the total protein of SH-SY5Y cells after 5-Aza-dC 48 h treatment. Rabbit anti-DJ-1 antibody (1:1000, sigma) were used for immunoblotting. The results were analyzed in three independent experiments.

### Statistical analysis

Statistical analysis was performed using SPSS13.0 and *P* < 0.05 was considered as significant. Statistical analysis included t tests for age. One-way ANOVA was used to evaluate the differences in mRNA and protein levels among SH-SY5Y cells treated by AZA, DMSO and mock groups.

## Results

1. Demographic characteristics
  The clinical characteristics are listed in Table 2. Each group comprised 24 men and 16 women, and there was no significant difference in age (*P* = 0.228) between the PD patients (63.7 ± 6.16 years) and NC (61.28 ± 9.21 years). Among the PD patients, 28 had mild degree of the disease (H &Y stage 1–2), 8 moderate (H& Y stage 3), and 4 severe (H&Y stage 4–5). Disease duration of 40 PD patients was 5 ± 2.7 years.

**Table 2:** Clinical characteristics of subjects

	NC (*n* = 40)	PD (*n* = 40)	*P*-Value
Age, years mean ± sd(lowest,highest)	61.28 ± 9.21(59,80)	63.7 ± 6.16(45,83)	0.228
Gender (male/female)	24/16	24/16	1.0
H&Y stage			
1–2 3 4-5	–	28	–
	–	8	–
	–	4	–
Duration, years mean ± sd(lowest,highest)	–	5 ± 2.7(2,14)	–
2. DNA Methylation of DJ-1 promoter region detected by Bisulfite specific PCR-based sequencing method
  Based on the NCBI database, the promoter region of human DJ-1 gene (NM_001123377) has two CpG islands (CGIs), locating at−1545 ~−1244 bp(CpG1),−1178 ~−732 bp(CpG2) upstream of the translation initiation site (Fig. 1a). CpG1 has 15 CpG sequences (Fig. 1b, a). CpG2 contains 21 CpG sequences (Fig. 1b, b). Bisulfite specific PCR-based sequencing method was used to examine the average methylation level at each CpG site (See methods). Our results showed that all the CpG sites probed in the two CGIs were unmethylated in both PD and NC (Fig. 2). The stable unmethylated status of CpG sites in both PD and NC group indicated that CpG methylation in the promoter region of DJ-1 in PBLs might have very limited or no regulatory effects on DJ-1 expression.

**Fig 1:**
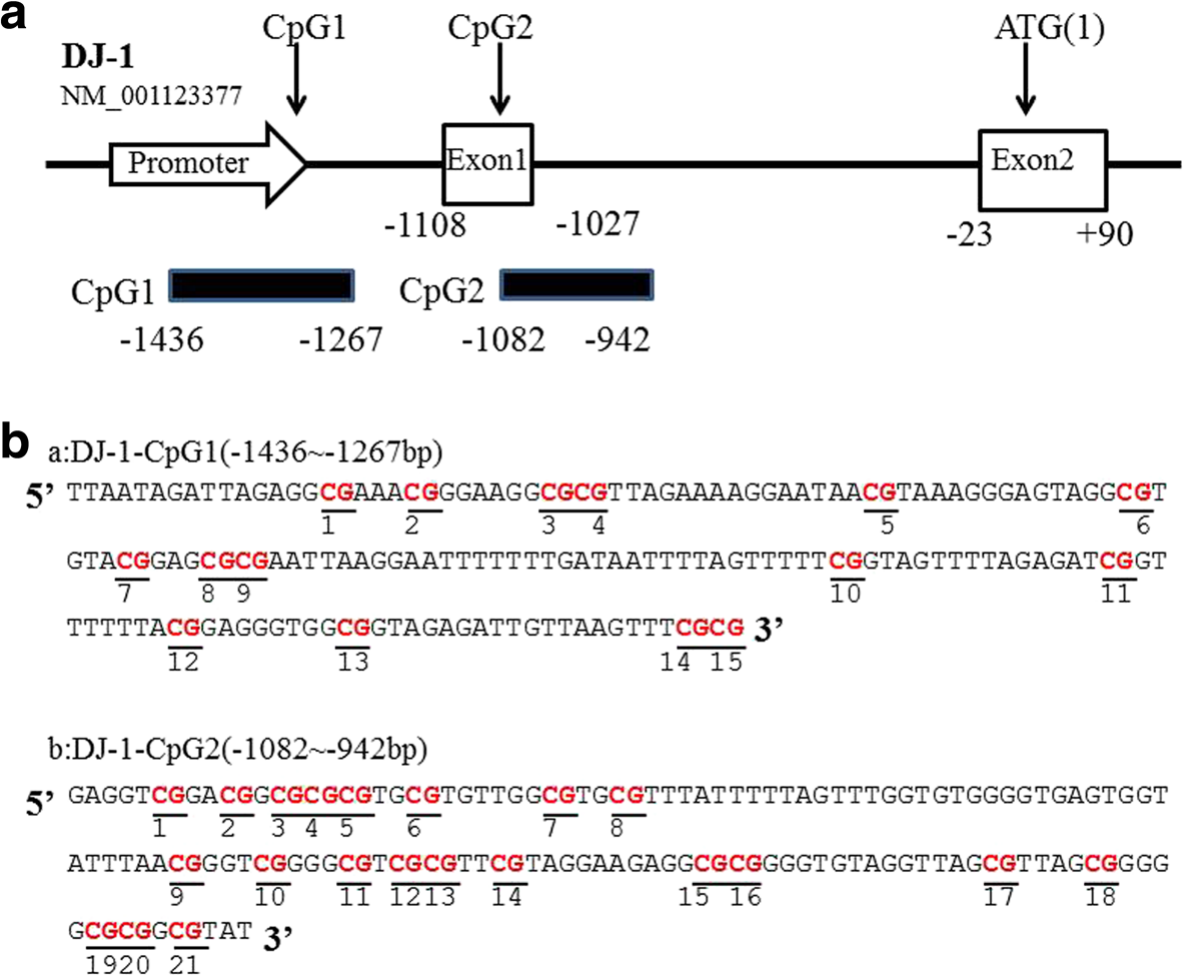
The two CpG islands in DJ-1. **a** Location of two CpG islands in DJ-1 (NM_001123377). **b** Sequence of CpG-1 islands in the promoter region of DJ-1(a), sequence of CpG-2 islands in exon 1 of DJ-1(b)

**Fig 2:**
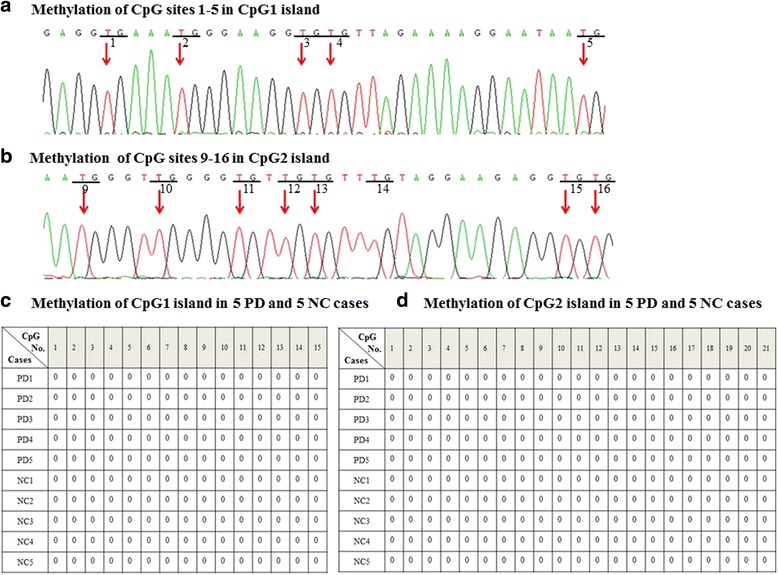
Bisulfite specific PCR-based sequencing analysis of two CpG islands methylation in 40 PD patients and 40 normal controls. **a** and **b** Unmethylated cytosines (**c**) were converted to uracil (U) after bisulfite treatment and amplified as thymines (T) in PCR amplication. Unmethylated CpG sites 1–5 in CpG1 island and CpG sites 9–16 in CpG2 island were shown here as an example. **c** and **d** All the CpG sites of these two CpG islands were unmethylated. Here are the methylation of CpG1 island and CpG2 island from 5 PD and 5 NC cases, and the rest cases showed the exact same methylation pattern
3. No effects of methylation inhibitor on the DJ-1 expression in SH-SY5Y cell model
  Previous whole genome bisulfite sequencing (WGBS) study showed that DNA methylation can be detected in various regions, including CGIs, gene bodies and tandem repeating-containing regions [15]. Moreover, methylation levels in these regions can regulate the transcription levels [15, 16]. Although the CpG sites probed in this study were unmethylated in both PD and NC group, the other CpG sites out of the promoter region haven’t been determined. In order to test whether CpG methylation level can regulate the DJ-1 expression, methylation inhibitor 5-Aza-dC was used to demethylate the CpG sites in a SH-SY5Y cell model, then DJ-1 expression was detected in the mRNA and protein level. Our results showed DJ-1 expression didn’t have significant change in both the mRNA and protein level in SH-SY5Y cells treated with 2.5 mM or 5 mM 5-Aza-dC compared with the untreated cells (Fig. 3). Thus, the results indicated that DNA methylation had no regulatory effects on DJ-1 expression in SH-SY5Y cell model.

**Fig 3:**
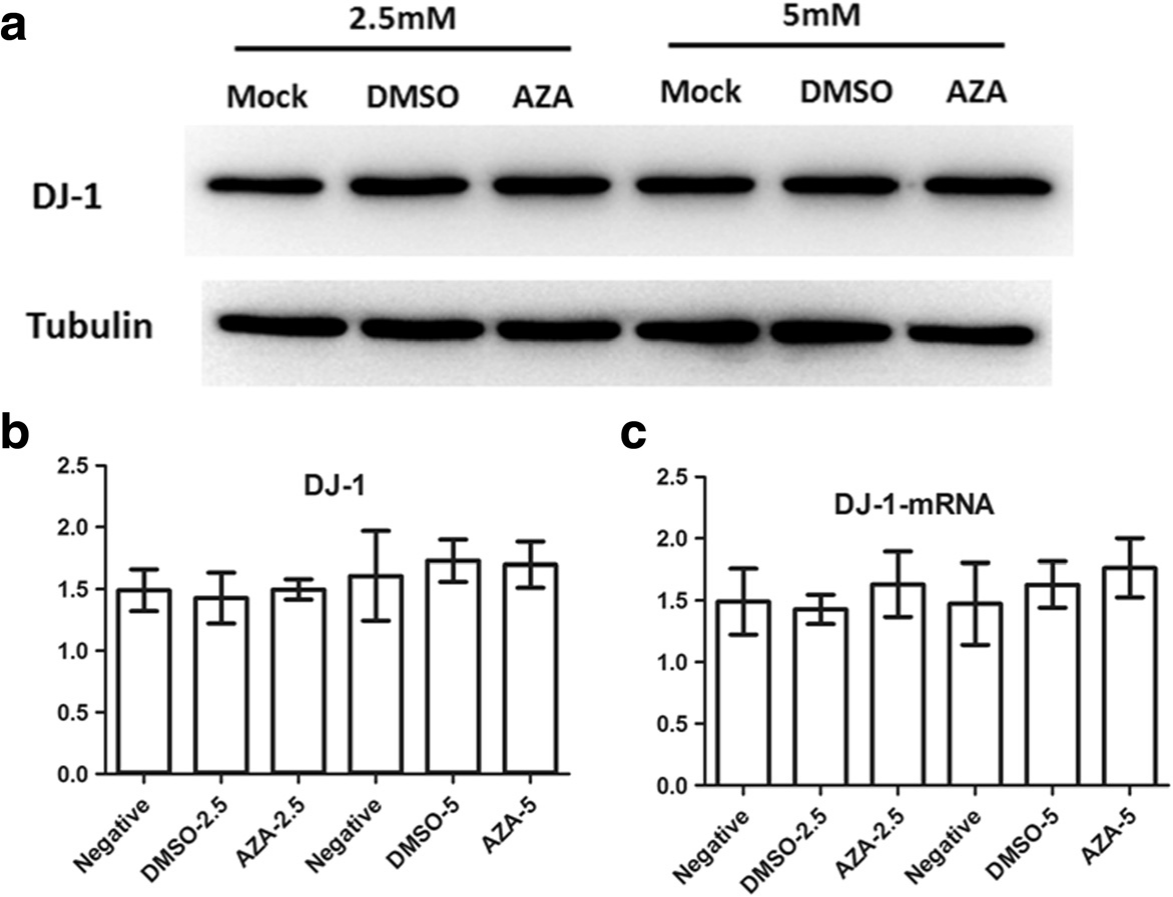
The mRNA and protein levels of DJ-1 did not change in 5-Aza-dC treated SH-SY5Y cells. Western blot and RT-PCR showed no significant change of DJ-1 protein level (**a** & **b**) and mRNA level (**c**) among 5-Aza-dC treated, DMSO treated and mock cells. Bar plot indicated the statistical analysis of DJ-1 protein level (**b**) and mRNA level (**c**) (Mean ± SD, *p < 0.05)

## Discussion

PD can begin years before the appearance of clinical motor symptoms when a significant number of nigrostriatal dopaminergic neurons have already been degenerated. The detection of PD prior to the emergence of motor symptoms is important for early diagnosis, neuroprotective treatment, monitoring disease progression and response to therapy. Thus, reliable biomarkers, which can represent a pathological process and can be easily detected, are needed. DJ-1 detected from brain [3], CSF [4, 5] and saliva [17] has been thought as a candidate biomarker of PD [18]. Recently, blood-based DNA methylation represents a highly promising biomarker for PD [13, 14] by regulating the causative gene expression. Thus, in the present study, we explored whether there was different DNA methylation level of DJ-1 in the PBLs between PD patients and NC.

Our results showed that in the two CGIs that we probed, all the CpG sites were unmethylated in both PD group and NC group, indicating that CpG methylation in the promoter region of DJ-1 in PBLs might have very limited or no regulatory effects on DJ-1 expression and could not be used as a biomarker to reflect DJ-1 expression changes in brains of PD patients and NC. However, previous WGBS study showed that DNA methylation can be detected in various regions, including CGIs, gene bodies and tandem repeating-containing regions [15]. Methylation levels in these regions can also regulate the transcription levels [15, 16]. Bisulfite specific PCR-based sequencing method used in our study only favored those CpG sites contained within CGIs and promoter regions [16]. Thus all other CpG sites out of the CGIs and promoter region can’t be probed by this method. In order to confirm whether CpG methylation can regulate the DJ-1 expression, we used methylation inhibitor to treat SH-SY5Y cells, then DJ-1 expression was detected in the mRNA and protein level. Our results revealed that there was no significant expression change of DJ-1 between the cells treated with methylation inhibitor and non-treated cells. Thus, our results indicated that DNA methylation might not be the key factor in regulating DJ-1 expression.

Generally, gene expression can be regulated in three different levels, transcriptional, mature mRNA processing and translational level. In the transcriptional level, both Sp1 and X-box-binding protein-1S (XBP-1S) were identified as transcription regulatory factor interacting with the DJ-1 promoter, thereby enhancing its promoter trans-activation, mRNA levels and protein expression [19, 20]. In mRNA processing level, miR-494 was found to decrease DJ-1 expression by binding to the 3’UTR of DJ-1 [21]. Till now, the epigenetic mechanism in modulating DJ-1 expression has not been fully explored. Zhou W et al. reported that phenylbutyrate, a HDAC inhibitor, can up-regulate the DJ-1 protein, indicating that DJ-1 expression can be regulated by histone modification [22]. In our study, we explored the possibility that whether DJ-1 expression can be regulated by the DNA methylation. We did not find differential CpG sites methylation in the CGIs of DJ-1 promoter region between PD and NC group, and there was no significant change of DJ-1 expression in a SH-SY5Y cell model treated by methylation inhibitor.

However, there were some limitations in our study. First, the sample size is small, and brain tissue as a standard control is not available in our country. Second, bisulfite specific PCR-based sequencing method used in our study is a convenient and economic method for DNA methylation screening, but it is a semi-quantitative method and only favored those CpG sites in the CGIs and promoter region [16]. Usually, we use PCR-based sequencing method for screening, if it provides any clue of different methylation, we would use bisulfite specific cloning-based sequencing, which is a more accurate and sensitive method to test the cytosine methylation at each CpG site in individual molecules [23], for further study. In this study, no methylated CpG sites were found in both PD and NC. Thus, the results would be the same even bisulfite specific cloning-based sequencing was used. As bisulfite specific PCR-based method only favors those CpG sites in CGIs and promoter region, in order to detect any regulatory effect of CpG sites methylation out of CGIs and promotor region on DJ-1 expression, we treated SH-SY5Y cells with methylation inhibitor and tested the DJ-1 expression level from both the mRNA and protein level, and our results confirmed no regulatory effects of DNA methylation on DJ-1 expression. In this study, we tried our best to get the truth, but the objective limits still exist. For further study, WGBS would be a good way to obtain an unbiased and more complete representation of the methylome by testing in both human brain tissue and PBLs.

## Conclusions

CpG sites are unmethylated in DJ-1 promoter region of PBLs from both PD patients and NC. DNA methylation inhibitor didn’t alter DJ-1 gene expression in SH-SY5Y cell model. Our results indicated that methylation status of DJ-1 had no obvious regulatory effects on DJ-1 expression, and might not be an efficient biomarker for PD patients.
